# Sexual Function and Associated Factors in Postmenopausal Women: A Systematic Review and Meta‐Analysis

**DOI:** 10.1002/hsr2.71270

**Published:** 2025-10-06

**Authors:** Mohadese Hosseinabadi, Najmeh Khodadadi, Hadi Tehrani, Arezoo Orooji, Seyedeh Belin Tavakoly Sany

**Affiliations:** ^1^ Social Determinants of Health Research Center Mashhad University of Medical Sciences Mashhad Iran; ^2^ Department of Health, Safety, and Environment Management, School of Health Mashhad University of Medical Sciences Mashhad Iran; ^3^ Department of Health Education and Health Promotion, School of Health Mashhad University of Medical Sciences Mashhad Iran; ^4^ Department of Biostatistics, School of Health Mashhad University of Medical Sciences Mashhad Iran

**Keywords:** Health Education, menopausal women, sexual dysfunction, sexual function, Women Health

## Abstract

**Background and Aims:**

The sexual health of postmenopausal women is frequently neglected, resulting in the common manifestations of menopause that lead to sexual dysfunction remaining unaddressed. This study aimed to systematically review the status of sexual function and dysfunction, and its association with modulating factors; and to examine the effectiveness of interventions in improving the status of sexual function in postmenopausal women.

**Methods:**

This systematic review and meta‐analysis study was conducted based on the preferred reporting items for systematic reviews and meta‐analyses (PRISMA) from inception until March 2022. Articles were searched through five electronic national and international databases including PubMed, Scopus, Scientific Information Database, Google Scholar, and Web of Science using the medical subject heading (MeSH) keywords related to the term “menopause”, “sexual function”, “sexual dysfunction”, and “menopausal women”.

**Results:**

Thirty‐seven studies with data from 9982 participants were eligible for the review, and 75% of the total studies reported sexual dysfunction among postmenopausal women. Age and education level significantly affected the risk of sexual dysfunction in women experiencing menopause. In the meta‐analysis of 21 cross‐sectional studies with 9262 participants found an overall effect size (ES) of 20.12 (95% CI: 17.39–22.86) for average sexual function and 0.65 (95% CI: 0.63–0.66) for the average percentage of sexual dysfunction among postmenopausal women. The pooled effect of the intervention on the average difference between the intervention and control groups was 3.08 (95% CI: 2.68–3.49), which was significantly different from zero (*p* < 0.001).

**Conclusion:**

This review reveals a significant prevalence of sexual dysfunction among postmenopausal women. The findings underscore the significant impact of educational interventions on enhancing sexual function in menopausal women. Consequently, it is advantageous to incorporate tailored intervention programs into medical education and healthcare practices to address sexual dysfunction during this challenging phase.

## 1 Introduction

Menopause is a natural event and complex period in a continuum of life stages for women and can affect physical, mental, emotional, and social well‐being among women [1]. Menopause is a “normal condition involving the permanent end of menstrual cycles due to the cessation of the production of reproductive hormones from the ovaries for at least 12 consecutive months” [2, 3]. During this time, women experience a range of complex symptoms due to both psychological and physical changes can lead to decreased sexual satisfaction in various aspects of life, including sexual health. Common symptoms include hot flashes, night sweats, heart palpitations, mood changes, vaginal dryness, anxiety, and insomnia [4, 5]. Addressing the level of sexual satisfaction during menopause involves understanding the multifaceted nature of the experience influencing sexual function. It is crucial to consider both the physical and emotional changes and interpersonal relationships that contribute to a woman's overall satisfaction during this life stage.

Changes in sexual function are a primary health concern after menopause, potentially leading to sexual dysfunction during this phase [2, 6]. Female sexual dysfunction is typically characterized by significant disturbances in sexual desire and physiological changes that can negatively impact the sexual response cycle, leading to substantial interpersonal difficulties and distress [7, 8]. Female sexual dysfunction is associated with various disorders, including sexual aversion, hypoactive sexual desire, sexual arousal and orgasm difficulties, and pain experienced during sexual activity [9]. To effectively evaluate sexual dysfunction, it is essential to ascertain whether a woman is sexually active and to identify any concerns related to orgasm, arousal, or pain experienced during sexual activity [3, 10].

Over the past 30 years, several studies have been conducted, yet none have been able to quantify the overall rate of sexual function in postmenopausal women. According to critical review research, in 2004, rates of sexual dysfunction were found to range from 1% to 75% for dyspareunia, 4% to 48% for arousal disorders, 1% to 50% for desire disorders, and 3% to 50% for orgasmic disorders [11] The 2008 review from Aslan et al. reported that rates of sexual dysfunction ranged from 68% to 86.5% [12, 13]. Last review in 2016, results showed that sexual dysfunction affects 41% of premenopausal women around the globe [14]. As studies have shown, the overall prevalence rate of sexual dysfunction during menopause varies significantly worldwide due to the complex nature of sexual function and the lack of globally standardized assessment tools.

Therefore, it is a difficult task to measure sexual function in women, as well as to establish meaningful steps in the intervention program. Currently, the most intervention programs are used to improve various aspects of sexual function for women experiencing menopause by combining education, psychological support, physical health, sexual therapy, and medical options [15, 16, 17, 18, 19, 20]. Although such intervention programs can to some extent, improve sexual function during this time, it remains unclear which types of intervention programs provide more benefit for postmenopausal women with sexual dysfunction. likewise, adequate evidence is not available to support the effectiveness of these intervention programs in practice.

Several studies have explored factors affecting sexual function in menopausal women, focusing on personal, social, and mental‐emotional aspects [21, 22, 23, 24, 25]. Personal factors include age, physical activity, health status, lifestyle, hormonal changes, menopause duration and type, the severity of symptoms, husband's sexual issues, and hormone therapies [26]. Social factors encompass occupation, socioeconomic status, religious beliefs, education, sexual knowledge, marriage duration, cultural background, social expectations, access to healthcare, and substance abuse. Mental‐emotional factors like anxiety, depression, body image, self‐esteem, and feelings about partners are also significant in understanding these issues [27]. Despite the existence of several studies assessing the overall effect of different factors associated with sexual function in postmenopausal women, as well as others that assess sexual dysfunction occurring in this period, several controversies still exist related to the direct effect of menopause on sexual function in different populations [28, 29]. Because of the complex nature of sexual function in postmenopausal women, addressing the factors primarily responsible for the disorder has never been an easy task [30]. To our knowledge, studies that systematically investigate the associated factors that affect sexual function in postmenopausal women are not been found in the literature. It is also unclear which personal, social, and mental‐emotional aspects may influence sexual function among postmenopausal women, and which intervention has better effectiveness in improving sexual dysfunction during menopause [10, 12]. Therefore, it is feasible to estimate the status of sexual function among postmenopausal women and to examine the factors that might affect sexual dysfunction.

To the best of our knowledge, there has not been a comprehensive review assessing the sexual function and associated factors among postmenopausal women across different populations. Therefore, we aim to systematically review the status of sexual function in postmenopausal women globally and to examine factors that might affect sexual dysfunction during menopause. Additionally, we intend to evaluate the effectiveness of educational interventions that may improve sexual dysfunction in women who have experienced menopause. This analysis will be valuable not only for statistical purposes but, more importantly, for its clinical relevance in finding potential solutions for sexual dysfunction.

## 2 Methods

The protocol of this study was already reviewed and approved by the Ethics Committee of Mashhad University of Medical Sciences (#IR. MUMS. REC.1401.096) after obtaining the required permit for the research. All procedures performed in studies in accordance with the ethical standards of the institutional research committee with the 1964 Helsinki declaration. Available https://ethics.research.ac.ir/form/da28jorie55g1432.pdf. We planned a systematic review and meta‐analysis based on the preferred reporting items for systematic reviews and meta‐analyses (PRISMA) [31] to address the following research questions:

- What is the status of sexual function in menopausal women in different countries?
- Which factors are associated with sexual dysfunction in postmenopausal women?
- Can health educational intervention improve the status of sexual function in postmenopausal women?

### 2.1 Search Strategy

Articles were searched using five international electronic databases, including PubMed, Scopus, Google Scholar, and Web of Science. The search utilized Medical Subject Headings (MeSH) keywords related to “menopause,” “sexual function,” “sexual dysfunction,” and “menopausal women.“The selection of databases is based on their extensive coverage and contributions to high‐quality research findings regarding sexual function in postmenopausal women, ensuring that the study is valid and scientifically sound. Two independent researchers conducted this search without a time limit until March 2022. Finally, the references in all selected articles were manually searched to identify additional studies that may have been overlooked due to the search terms used.

### 2.2 Screening and Selection

Table S1 shows the details related to the inclusion and exclusion criteria based on the PICOS index. Initially, two independent authors screened all retrieved articles by reviewing their abstracts, titles, and full texts. Articles were included in this review if they met the inclusion criteria, and both authors agreed 100% on the eligibility of these articles. Further, if there was any uncertainty about an article's eligibility, any ambiguities or discrepancies were evaluated by a third author.

### 2.3 Extraction and Quality Control

Data extraction was conducted via a pre‐designed form of meta‐analysis and systematic review based on the following information: (1) author's name, year of publication, study location and design, participant characteristics, sample size, and objectives; (2) sexual function scores and percentage of sexual dysfunction; (3) setting and type of tools used to measure sexual function; (4) type of intervention, sample size, method analysis, duration, effectiveness, condition of control, intervention, and follow‐up, and effect size, and (5) estimation of the association between sexual function/dysfunction and social‐demographic characteristics based on p values. To conduct quality control, first, the eligibility of full‐text articles was independently assessed based on the inclusion and exclusion framework. Second, an independent dual rating was conducted to evaluate the quality of both cross‐sectional and interventional studies using the JBI critical appraisal checklist [32, 33] and and the Cochrane risk of bias tool [32, 34], respectively. All doubts and disagreements were resolved via discussion meetings.

### 2.4 Meta‐Analysis Assessment

In this review, the level of sexual function/dysfunction was considered as a main target in the meta‐analysis; therefore, all relevant articles with a validated measure of sexual function/dysfunction were included in the meta‐analysis.

In this review, most of the included studies utilized the long version of Female Sexual Function Index (FSFI) questionnaire to assess the level of sexual function. Consequently, only these studies were included in the meta‐analysis [1, 15, 17, 18, 19, 20, 21, 23, 24, 25, 28, 35, 36, 37, 38, 39, 40, 41, 42, 43, 44, 45, 46, 47, 48, 49, 50, 51, 52, 53, 54, 55, 56]. This selection increases the power of meta‐analyses to evaluate the overall status of sexual function and dysfunction using the same scale while reducing heterogeneity. This questionnaire typically has a total score that ranges from 2 to 36, with a commonly used cutoff score of 26.55 to identify women at risk for sexual dysfunction. The mean and standard deviation of the FSFI score were deemed appropriate for analyzing the effect size (ES) of sexual function.

Additionally, only 12 studies, which represent 32% of the total studies, defined sexual dysfunction using percentages. All these studies also specified the cutoff score of 26.55 for identifying those at risk of sexual dysfunction [16, 22, 28, 39, 42, 46, 47, 48, 50, 52, 53, 57]. Therefore, the percentage of the population based on the cutoff score of 26.55 was the unit of analysis for measuring the effect size of sexual dysfunction for these studies.

Likewise, the correlation coefficient (r) was used as the ES of the association because it is the most common unit of analysis of ES in meta‐analysis articles and shows both negative and positive directions or relationships between different variables. Thus, this study converted other ES such as linear regression coefficients, X^2^ (squared values of the predictor variables), and t‐test values to the “r” measure, while a negative “r” indicates that sexual function or dysfunction is inversely associated with social‐demographic factors; a positive “r” shows that sexual function or dysfunction is directly associated.

In Meta‐analysis, heterogeneity in ES with 95% confidence intervals was evaluated using a fixed‐effects model based on Thompson's I^2^ statistic and Q value. Likewise, the random effects model estimated the association between different variables. Thompson's I^2^ estimates the “I^2^ measures the proportion of total variation in study measures due to heterogeneity. The Q value checks whether the non‐variation of the effects is greater than the non‐variation due to sampling error”. If the p‐value for Q is lower than 0.05, heterogeneity is considered suitable for the interpretation of data. Further, we used meta‐regression analysis to estimate the effect of covariates on pooled effects and to find a suitable study for stratified analysis. The potential publication bias was evaluated using Egger's test and trim‐and‐fill via funnel plot asymmetry. Subgroup analysis was utilized to investigate specific subsets of sexual function or dysfunction within the included studies, aiming to identify outcome differences based on various study locations. STATA software version 14 (Corp LP) was run to analyze all relevant statistical tests.

## 3 Results

### 3.1 Search Outcome

**Figure 1:**
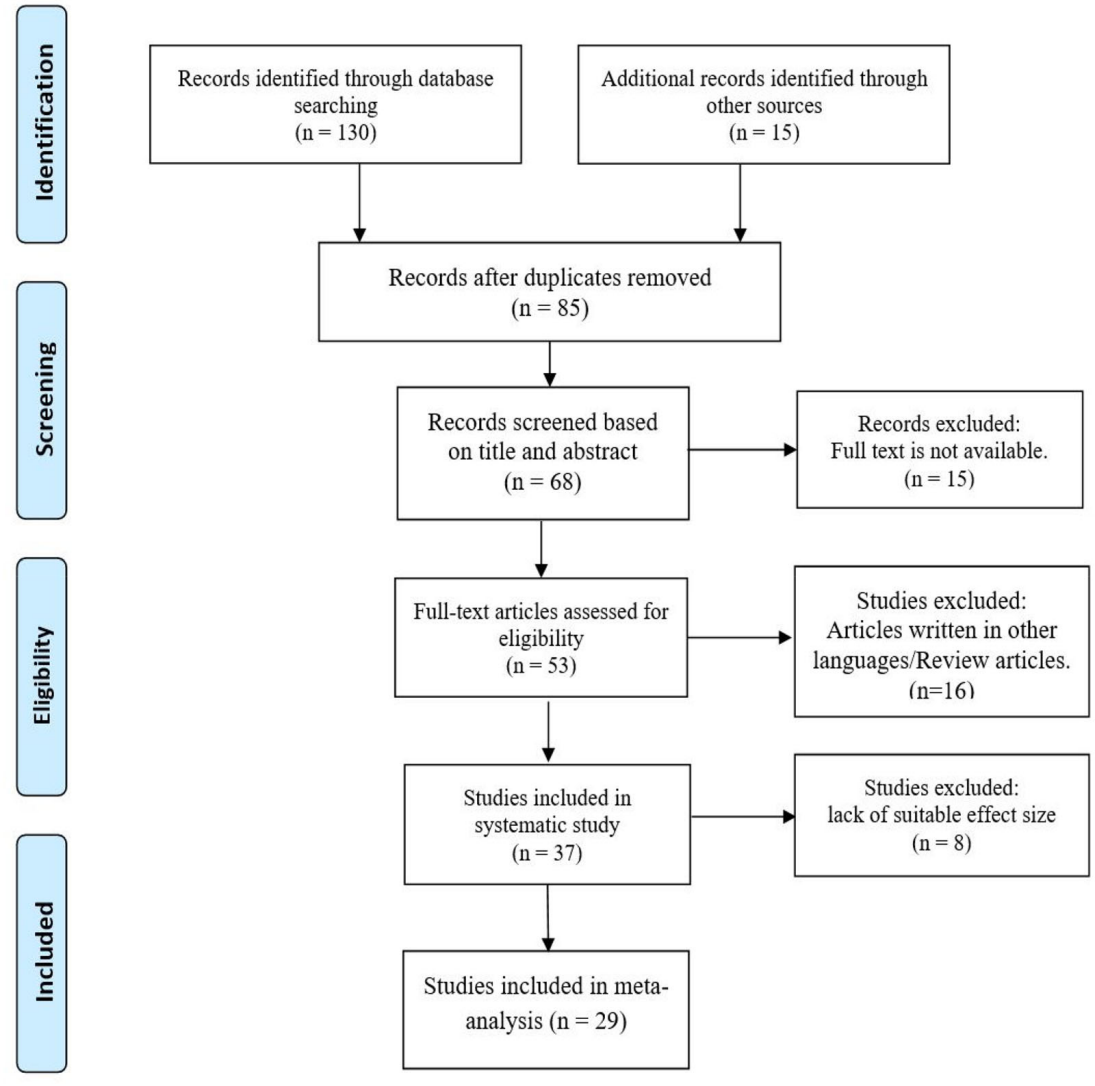
Studies selection process based on PRISMA.

A total of 130 studies were identified through database searches, and an additional 15 studies were found through other sources. After removing 60 duplicate articles, 68 articles were screened based on their titles and abstracts. Of these, 15 articles were excluded due to the unavailability of their full texts. The full texts of the remaining articles were then evaluated for eligibility, leading to the exclusion of 16 studies because they were either review articles or written in languages other than English. Ultimately, 37 studies with data on 9982 participants were selected for the systematic review [1, 15, 16, 17, 18, 19, 20, 21, 22, 23, 24, 25, 28, 35, 36, 37, 38, 39, 40, 41, 42, 43, 44, 45, 46, 47, 48, 49, 50, 51, 52, 53, 54, 55, 56, 57, 58]. Of these, 8 studies were excluded due to the lack of a suitable effect size, resulting in 29 studies with data on 9262 participants being included in the meta‐analysis [1, 21, 22, 23, 24, 25, 28, 35, 36, 37, 38, 39, 40, 41, 42, 44, 45, 46, 47, 48, 49, 50, 51, 52, 53, 54, 55, 57, 58] (Table 1 and Figure 1).

**Table 1:** Study samples characteristics based on articles published until March 2022.

Code	Authors	Country	Age (years)	Education (%)	Income (%)	Sample size	menopause Duration (months)	Sexual function (m ± SD)	Dysfunction (%)
1	Marván,2017	Mexico	53.4 ± 3.6	Under Diploma: 38.80, Diploma and higher: 61.22	—	253	—	13.65 ± 4.93	—
2	Jamali,2015	Iran	60.10 ± 6.89	Illiterate: 36.71, Under diploma: 46.5, Diplom and higher: 16.83	—	746	96 ± 72.11	19.31 ± 9.69	81.51
3	Hashemi,2013	Iran	53.11 ± 4.56	—	—	225	60.97 ± 72.40	—	51.33
4	Eftekhar,2016	Iran	< 50(70%), > 50(30%)	Under Diploma: 39, Diploma and higher: 61	—	151	< 10: (1%), 10–20:(28%) > 20: (72%)	10.5 ± 11.16	53
5	Nazarpour,2015	Iran	52.84 ± 3.71	Illiterate: 63.51, Under Diploma: 27.75, Diploma and higher: 8.90	—	405	19.81 ± 14.36	24.11 ± 6.04	—
6	Bostani,2019	Iran	59.13 ± 1.25	UnderDiploma: 55.12, Diploma and higher: 44.88	—	215	—	—	36.28
7	Beigi,2012	Iran	54.67 ± 6.30	—	—	174	—	—	72.40
8	Omidvar,2011	Iran	55.92 ± 6.02	Under Diploma: 95, Diploma and higher: 5	Good (68.90) Moderate (14.30)	280	84.50 ± 60.43	—	56.41
9	Chedraui,2010	Spain	45	Under Diploma: 23.32, Diploma and higher: 76.71	—	262	—	28.70	—
10	Lett,2017	Brazil	54.13	—	—	540	< 60: 45.6% 60–120: 51% ≥ 120: 59.6%	—	50.62
11	Herrezuel,2020	Spain	65.59 ± 7.93	Under Diploma: 59.89, Diploma and higher: 39.56	—	182	—	18.22 ± 10.62	—
12	Wong,2012	Hong Kong	58 ± 5.01	Under Diploma: 68.90, Diploma and higher: 31.11	Good (47.90) Moderate (52.20)	540		—	10.1 ± 10.3
13	Alves,2015	Brazil	55 ± 4	—	—	184	108 ± 60	18.14 ± 11	1D:27, T2D:85
14	Jonusiene,2009	USA	55.50 ± 5.22	—	—	246	—	22.6 ± 6.64	67.91
15	Dundo,2010	USA	52.00 ± 6.05	—	—	86	—	18.48 ± 9.18	—
16	Avis,2017	USA	46.33 ± 2.60	Under Diploma: 24.22, Diploma and higher: 75.88%	Low (6.30), Moderate(30.50), Good (63.20)	1390	—	18 ± 3.49	—
17	Ornat,2012	Spain	40–44(35.4%) 45–49(31.9%) 50–54 (25%) 55–59 (7.7%)	Illiterate: 0.80, UnderDiploma: 85, Diploma and higher: 14.20		260	—	—	39.71
18	Alizadeh,2012	Iran	54.22 ± 2.80	Illiterate: 57, Under Diploma: 41.80, Diploma and higher: 2.20	Not enough: 49.55	400	—	53.3 ± 29.32	—
19	Jafarbegloo,2012	Iran	54.34 ± 6.54	—	—	119	—	22.35 ± 8.87 (82.4%)	—
20	Peixoto,2019	Brazil	55.39 ± 4.68	Study(Y): 7.33 ± 4.49, [min=0; max=17]	min=300.00 to max=4000.00	36	—	13.31 ± 9.15	—
21	Gozuyesil,2016	Turkey	49.52 ± 6	Under Diploma: 81.10, Diploma and higher: 18.90	—	317	—	18.8 ± 8.79	—
22	Alavipour,2018	Iran	54.12 ± 4.25	Under Diploma: 81.60, Diploma and higher: 18.40	Low:19.37 Moderate :59.05 High:21.59	315	65.82 ± 47.55	18.92 ± 4.25	—
23	Nazarpour,2015	Iran	52.84 ± 3.74	Illiterate: 63.50, Underdiploma: 27.70, Diploma and higher: 8.90	—	405	19.81 ± 14.36	24.11 ± 6.04	61
24	Afshari,2015	Iran	45–50(50%) 51–55(27%) 56–60 (23%)	Illiterate: 17, Under diploma: 83,	Moderate: 93 Poor: 7	437	12–60: 36%, 72–120: 14% > 132: 3%	17.93 ± 5.44	—
25	Moghassemi 2008	Iran	52.19 ± 3.76	Illiterate: 4.10, Under diploma:85.70, Diploma and higher:10.10	—	149	13.21 ± 1.53	21.13 ± 6.02	86.62
26	Golzari,2019	Iran	52.92 ± 5.34	Under diploma: 86, Diploma and higher: 14	Satisfied: 27.9 Unsatisfied:72.1	258	—	28.09 ± 4.48	—
27	riazi,2017	Iran	55.36	Under Diploma: 94, Diploma and higher: 6	Good:18 Moderate:68 Poor:14	50	—	22.43 ± 7.32	—
28	Afshari,2015	Iran	45–50(50%) 51–55(27%) 56–60(23%)	Illiterate: 17, Under diploma: 83	Moderate: 93 Poor: 7	437	12–6: 36% 72–120: 14% > 132: 3%	19.28 ± 7.26	—
29	Alirezaei,2017	Iran	57.18 ± 7.93	Under diploma: 83.50, Diploma and higher: 16.50	Enough: 72.5 Poor: 27.5	200	7.80 ± 7.00	22.53 ± 5.91 (27.5%)	—
30	Alves,2011	Brazil	52.13 ± 3.52	—	—	32	12–60	69.3 ± 20	—
31	Nazarpour,2015	Iran	53.13 ± 2.67	Under diploma: 51.10, Diploma and higher: 48.90	—	97	23.87 ± 16.20	23.52 ± 0.55	—
32	Mirmohamad, 2015	Iran	47–49(13.34) 50–52 (37.82%) 53–55 (48.91%)	Under diploma: 82.20, Diploma and higher: 17.80	—	100	12–60	2.47 ± 4.52	—
33	Nazarpour,2015	Iran	G1:51.5 ± 3.42,G2:53.11 ± 2.70	Under diploma: 82.20, Diploma and higher: 17.80	—	145	—	SE:24.50 ± 0.50 K:23.81 ± 0.51	—
34	Nazarpour,2016	Iran	51.54 ± 3.35	Under Diploma: 47.90, Diploma and higher: 52.10	—	104	20.85 ± 16.20	24.41 ± 0.52	—
35	Naeij,2017	Iran	51.53 ± 3.34	Under Diploma: 58, Diploma and higher: 42	Low: 38 Enough: 62	52	3.50 ± 1.55	28.20 ± 4.43	—
36	Alavipour,2019	Iran	55.17 ± 4.54	Under diploma: 84.40, Diploma and higher: 15.60	low:15.6, Moderate: 68.9 Satisfied:15.6	90	77.80 ± 64.91	23.70 ± 3.67	—
37	Ghelichkhani, 2014	Iran	47–49(12%) 50–52(40%) 53–55(48%)	Unde rdiploma: 82, Diploma and higher: 18	—	100	12–60	22.09 ± 4.25	—

All studies used long version of Female sexual function index (FSFI) questionnaire to measure sexual function except studies 10, 17, 18, and 30 and 32 that used SPEQ, CSFQ‐14, MFSQ, and SQ‐F, respectively; SE (Sex education) K (Kegel exercises); studies with code 30 to 37 is related to experimental studies; United states of America (USA). Under diploma” refers to an educational programs are at a lower level than a diploma such as high school and lower grades. Diplomas is the the first degree after high school, but below a bachelor's degree. Higher diploma refers academic degrees given by a university such as ba bachelor's and master's dgree

All the data were collected from 7 countries, including Iran, Brazil, the United States of America, Spain, Turkey, Hong Kong, and Mexico. The results showed that 34 studies (92%) were conducted in Iran (64% of total studies) [1, 15, 17, 18, 19, 20, 24, 25, 28, 35, 36, 37, 39, 42, 43, 44, 46, 47, 50, 51, 52, 53, 56, 58] Brazil (12%) [16, 22, 23, 54], USA (8%) [38, 41, 48], and in Spain (8%) [21, 40, 57]. Other studies were conducted in Turkey (2%) [45], Hong Kong (2%) [55], and Mexico (4%) [49]. The sample size ranged from 32 to 1390 [16, 38]. The initial studies were published in 2009 [41], with 64% of the included research being released between 2018 and 2022 [15, 17, 20, 21, 22, 23, 24, 28, 35, 36, 37, 43, 44, 49, 52, 54, 55] (Table 1).

### 3.2 Study Designs and Populations

Of the 37 studies included in systematic review, 29 studies are cross‐sectional studies (78% of total included studies) [1, 21, 22, 23, 24, 25, 28, 35, 36, 37, 38, 39, 40, 41, 42, 44, 45, 46, 47, 48, 49, 50, 51, 52, 53, 54, 55, 57, 58] (Table 1) and 8 articles are experimental studies (22%) [15, 16, 17, 18, 19, 20, 43, 56], which evaluated eight interventions programs based on personal exercise program (PEP) training, pelvic floor muscle (PFM) exercises, education sessions or booklets, sex counseling workshop, and DVD decision aids (Table S2). The average age and standard deviation of the population of postmenopausal women in 37 studied studies is equal to 54.01 ± 4.5 [1, 15, 16, 17, 18, 19, 20, 21, 22, 23, 24, 25, 28, 35, 36, 37, 38, 39, 40, 41, 42, 43, 44, 45, 46, 47, 48, 49, 50, 51, 52, 53, 54, 55, 56, 57, 58]. The minimum and maximum ages of the studied subjects are 45 and 65 years, respectively. The average education in these studies showed that 32.4% of people were illiterate, 64.2% had a diploma, and 28.5% had a diploma or higher. The analysis of the population's income levels shows that 43.9% reported having an adequate income and were satisfied with their income. Additionally, 56.8% fall within the moderate‐income category, while 14.2% are classified as low‐income [1, 16, 17, 18, 19, 20, 21, 22, 23, 24, 25, 28, 35, 36, 37, 38, 39, 40, 41, 42, 43, 44, 45, 46, 47, 48, 49, 50, 52, 53, 54, 55, 56, 57, 58] (Table 1).

### 3.3 Quality Assessment

**Figure 2:**
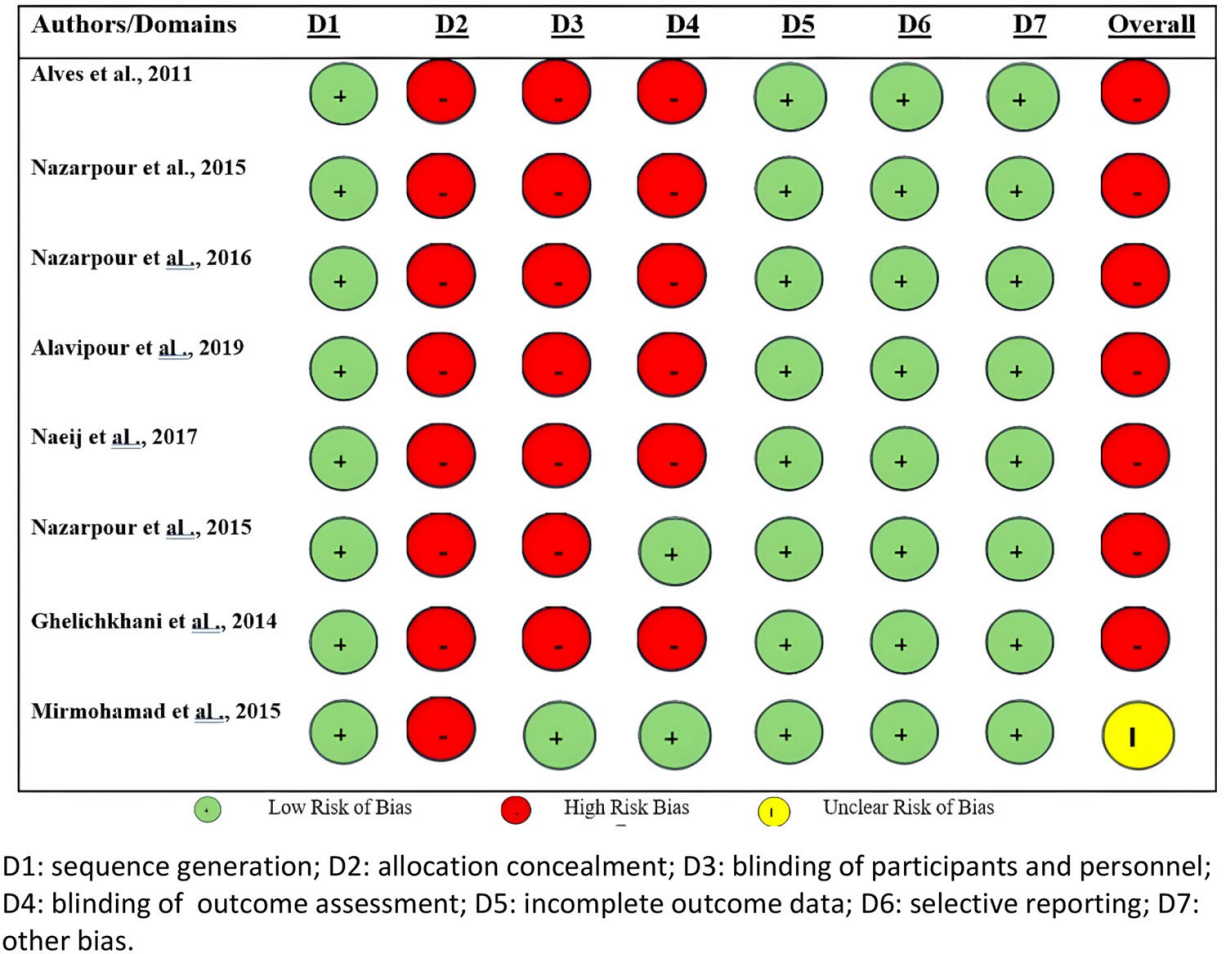
The Cochrane Collaboration's tool for assessing risk of bias in experimental studies D1: sequence generation; D2: allocation concealment; D3: blinding of participants and personnel; D4: blinding of outcome assessment; D5: incomplete outcome data; D6: selective reporting; D7: other bias.

The quality of included experimental studies was evaluated based on the Cochrane risk of bias tool. Of the 8 experimental studies, only the quality of one study was acceptable [43], and the quality of others studies was moderate [15, 16, 17, 18, 19, 20, 43, 56]. Almost all of the experimental studies did not clearly report allocation concealment (100%) [15, 16, 17, 18, 19, 20, 43, 56], blinding of outcome assessment (75%) [1, 15, 16, 17, 18, 19, 20, 21, 22, 23, 24, 25, 28, 35, 36, 37, 38, 39, 40, 41, 42, 43, 44, 45, 46, 47, 48, 49, 50, 51, 52, 53, 54, 55, 56, 57, 58], and the blinding of participants and personnel (87.5%) [15, 16, 17, 18, 19, 20, 56]. At the same time, all included studies reported selective reporting, outcome assessment, random sequence generation, and other biases (Figure 2).

The quality of the included cross‐sectional research was moderate. All the studies used a reliable and valid way to measure exposure and outcome, standard criteria for their measurement, and appropriate statistical analysis as well as clearly described the study subject and setting in detail [1, 21, 22, 23, 24, 25, 28, 35, 36, 37, 38, 39, 40, 41, 42, 44, 45, 46, 47, 48, 49, 50, 51, 52, 53, 54, 55, 57, 58]. Based on the JBI checklist, it was observed that 20 studies did not adequately describe confounding factors, while 27 studies failed to outline the strategies employed to address these factors [1, 21, 22, 23, 24, 25, 28, 35, 36, 37, 38, 39, 40, 41, 42, 44, 45, 46, 47, 48, 49, 50, 51, 52, 53, 54, 55, 57, 58]. Similarly, three studies did not clearly define the criteria for including participants in the sample [1, 15, 16, 17, 18, 19, 20, 21, 22, 23, 24, 25, 28, 35, 36, 37, 38, 39, 40, 41, 42, 43, 44, 45, 46, 47, 48, 49, 50, 51, 52, 53, 54, 55, 56, 57, 58] (Table 2).

**Table 2:** Critical appraisal JBI checklist to evaluate the quality of cross‐sectional studies.

Questions	Critical appraisal checklist	Yes		No	Unclear
1	Were the criteria for inclusion in the sample clearly defined?	26		3	0
2	Was the exposure measure in valid and reliable way?	29		0	0
3	Were objective, standard criteria used for measurement of the condition?	29		0	0
4	We're confounding factors identified?	25		20	0
5	Were the study subject and the setting described in detail?	29		0	0
6	Were strategies to deal with confounding factor stated?	2		27	0
7	Was the outcome measured in a valid and reliable way?	29		0	0
8	Was appropriate statistical analysis used?	29		0	0

### 3.4 Systematic Review

Our findings showed that the female sexual function index questionnaire (FSFI) was the most common questionnaire used to measure sexual dysfunction in postmenopausal women. Specifically, 33 studies (89% of total 37 studies utilized the FSFI to measure sexual dysfunction [1, 15, 17, 18, 19, 20, 21, 23, 24, 25, 28, 35, 36, 37, 38, 39, 40, 41, 42, 43, 44, 45, 46, 47, 48, 49, 50, 51, 52, 53, 54, 55, 56]. The remaining 11% of studies employed alternative questionnaires, including the Sexual Quotient‐Female Version (SQ‐F), the McCoy Female Sexuality Questionnaire [58], the Short Personal Experiences Questionnaire [22], and the 14‐item Changes in Sexual Functioning Questionnaire (CSFQ‐14) [57]. Existing measures of sexual function, such as FSFI, SQ‐F, and CSFQ‐14 are designed based on specific domains to assess desire, arousal, lubrication, orgasm, satisfaction, and pain to measure sexual function.

A total of 22 studies (75%) reporting sexual function based on the mean and standard deviation measured by the long version of FSFI. The level of sexual function was ranged from 10.5 ± 11.16 to 28.09 ± 4.48 (Table 1). The average sexual function in these studies was 21.54 ± 7.97, which is considered sexual dysfunction because the score was less than or equal to 26.55, indicating sexual dysfunction based on the FSFI measure (Table S3). A study on the sexual function domains showed a significant difference among the level of sexual function domains in 18 studies (55%) [1, 15, 17, 18, 19, 20, 24, 25, 28, 35, 36, 37, 39, 42, 43, 44, 46, 47, 50, 51, 52, 53, 56, 58]. Among them, the lowest score was correlated with the domains of arousal, desire, and orgasm, while the highest score was associated with the satisfaction and pain domain (Table S4).

Likewise, 12 studies (32%) expressed sexual dysfunction in the form of percentages [16, 22, 28, 39, 42, 46, 47, 48, 50, 52, 53, 57] from 27% to 86.6% of the total population in these studies. The overall average of sexual dysfunction is 56.97% in these studies. Nine studies reported sexual dysfunction above 50% [16, 39, 42, 46, 47, 48, 50, 52, 53], and 4 studies reported that sexual dysfunction is lower than 50% in their total population [16, 50] (Table 1).

As shown in Table 1, associations between sexual function and age were reported only in 9 studies [21, 22, 23, 24, 25, 40, 45, 48, 57], of which 8 studies showed significant negative associations between sexual function and age (*p* < 0.05) [21, 23, 24, 25, 40, 45, 48, 57]. Likewise, only 3 (8% of total studies) studies measured associations between sexual function and participants’ education, and they reported a significant positive relationship (*p* < 0.05) between participants’ sexual function and education [17, 21, 40]. In this review, 9 studies examined the relationship between sexual function and participants’ income [16, 39, 42, 46, 47, 48, 50, 52, 53]. Of these, 4 studies found significant positive associations between sexual function and income (*p* < 0.05) [39, 47, 48, 52]. However, 2 studies conducted in Iran reported a negative association between sexual function and women's income during menopause.

According to the available results, the association between sexual function with age and education has been measured in limited studies, and the relationship between sexual function and other socio‐demographic factors has not been investigated and evaluated. Since the results from these associations were reported in a limited number of studies [21, 22, 23, 24, 25, 40, 45, 48, 57], it is not possible to determine specific trends or draw conclusions about the impact of sociodemographic characteristics on levels of sexual function and dysfunction In postmenopausal women.

In this review study, 8 studies (21% of total studies) evaluated the effect of the intervention (PEP training, PFM exercises, training protoco, Kegel exercises, sex counseling workshop, and DVD decision aids) [15, 16, 17, 18, 19, 20, 43, 56] on improving the sexual function among postmenopausal women (Table S2). Of which, 4 studies showed a significant effect on improving the sexual function in the intervention group compared to the control group [15, 18, 20, 56] (Table S2).

### 3.5 Meta‐Analysis

#### 3.5.1 Sexual Function Status

In this review, 21 cross‐sectional study with data on 9262 participants have suitable effect size (ES) to evaluate sexual function and met inclusion criteria to enter the meta‐analysis [21, 22, 23, 24, 25, 28, 35, 36, 37, 38, 39, 40, 41, 42, 44, 45, 46, 47, 48, 49, 50, 51, 52, 53, 54, 55, 57, 58]. All these studies measured the level of sexual function based on the FSFI as most comment tool questionnaire [1, 15, 17, 18, 19, 20, 21, 23, 24, 25, 28, 35, 36, 37, 38, 39, 40, 41, 42, 43, 44, 45, 46, 47, 48, 49, 50, 51, 52, 53, 54, 55, 56]. This helps us to increase the power of meta‐analyses to measure the overall status of sexual function and dysfunction based on the same scale. The total score of included studies gave an overall ES of 20.12 (95% CI: 17.39–22.86) for the average sexual function among participants, suggesting sexual dysfunction based on the FSFI measure.

**Figure 3:**
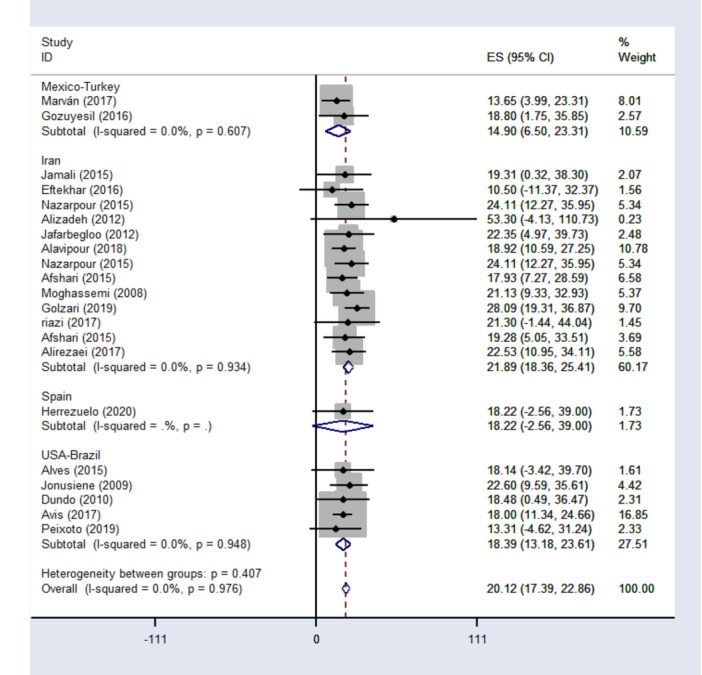
Forest plot of sexual function status in postmenopausal women, stratified by study location, ES: effect size, I^2^: a measure of the ratio of the total variation in study measures due to heterogeneity, *p* > 0.05: devastates heterogeneity is appropriate for interpretation.

In all the studies included in the meta‐analysis, the average score for sexual function was below the cutoff value of the Female Sexual Function Index (FSFI), which is set at 26.55. The only exception was Alizadeh's study, which presented a significantly higher average score of 33.30 (95% CI: 14.13–35.73), making it an outlier compared to the other studies. The reported pooled effect of sexual function in Mexico and Turkey, Iran, Spain, America, and Brazil are estimated to be 14.90 (95% CI: 6.5–23.31), 21.89 (95% CI: 18.36–25.41), 18.22(95% CI; 2.56–32.00), and 18.39 (95% CI; 13.8–23.61), respectively (Figure 3).

**Figure 4:**
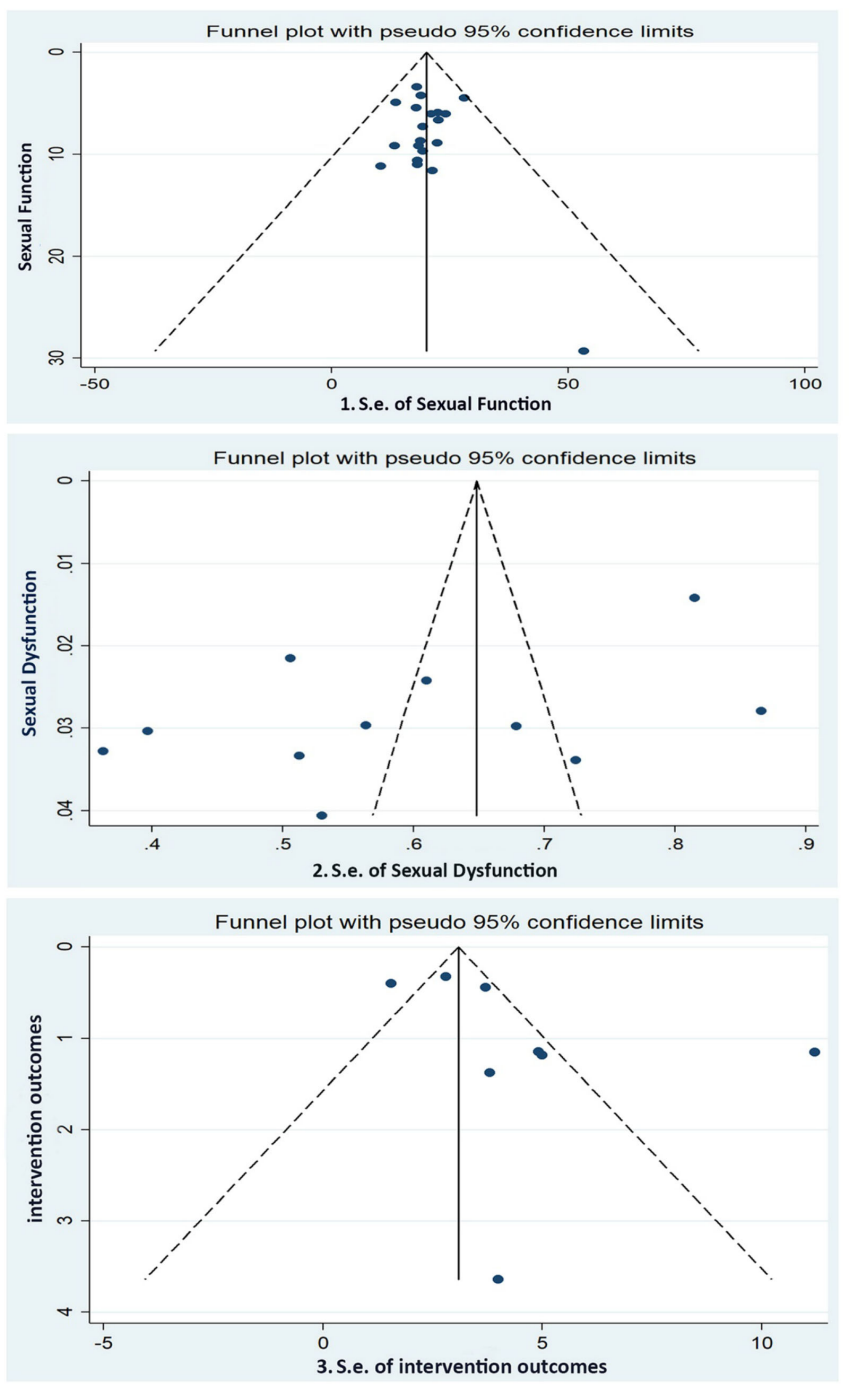
Funnel plot of standard error with peudo 95% confidence limit to assess publication bias for sexual function status, sexual dysfunction, and intervention outcomes.

As shown in Figure 3, the heterogeneity within each subgroup is zero, indicating no significant differences among the countries within each subgroup (*p* = 0.97). The I² statistic was also insignificant at 0.0% (*p* = 0.976) for the overall effect size (ES) of sexual function, demonstrating adequate homogeneity in the fixed‐effects results. Additionally, the funnel plot analysis revealed no significant deviations or gaps among the 21 studies at both the high and low ends of the range, indicating that the symmetry of the funnel plot is acceptable (Figure 4).

Egger's test was conducted to evaluate publication bias, and no significant evidence of bias (*p* = 0.74) was found in the assessed studies. Additionally, since the heterogeneity was found to be zero, and one of the aims of the studies was to explore the association between sexual dysfunction and sociodemographic factors, subgroup and meta‐regression analyses were performed. The meta‐regression analysis revealed that factors such as study location, study period, participants’ education level (0.32), age (*p* = 0.68), time of study (*p* = 0.07), and sample size (*p* = 0.067) did not contribute to heterogeneity and had no significant impact on the overall effect size (ES) of sexual function across all studies.

**Figure 5:**
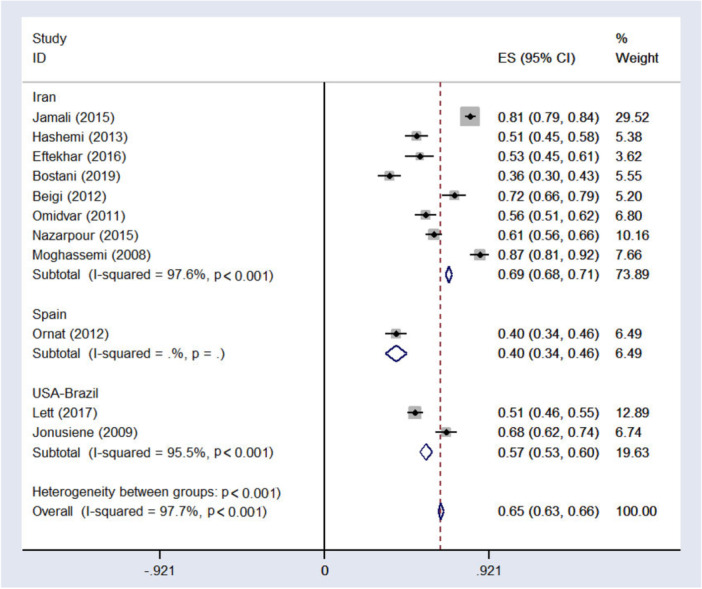
Forest plot of sexual dysfunction in postmenopausal women, stratified by study location, ES: effect size, I^2^: a measure of the ratio of the total variation in study measures due to heterogeneity, *p* > 0.05: devastates heterogeneity is appropriate for interpretation.

According to the above diagram, 11 studies met meta‐analysis criteria to measure the level of sexual dysfunction in different populations based on percentages of the total population. The total score of included studies gave an overall ES of 0.65 (95% CI: 0.63–0.66) for the percentage of sexual dysfunction among participants, suggesting that 65% of total participants had sexual dysfunction. The statistic was significant (97.7%, *p* < 0.001) for the overall ES of sexual dysfunction, indicating a heterogeneity within the fixed‐effects results **(**Figure 5).

Egger's test and the relevant funnel diagram were drawn, and the results of this test showed that publication bias did not occur (*p* = 0.057). However, due to the asymmetry of the funnel plot, the trim and fill method was also used to test the publication bias, and its result showed an insignificant publication bias (*p* = 0.064) (Figure 4).

The asymmetry observed in the funnel plot is likely caused by the studies conducted by Jamil, Bostan, Beigi, Omat, and Moghassemi, which significantly inflate the pooled effect measure for assessing the prevalence of sexual dysfunction across different populations. Meta‐regression analysis revealed that factors such as education level (*p* = 0.34), age (*p* = 0.73), sample size (*p* = 0.068), study location (*p* = 0.082) and time of study (*p* = 0.078) did not contribute to heterogeneity and had no significant impact on the overall effect size of sexual dysfunction across all studies.

#### 3.5.2 Intervention Effect

**Figure 6:**
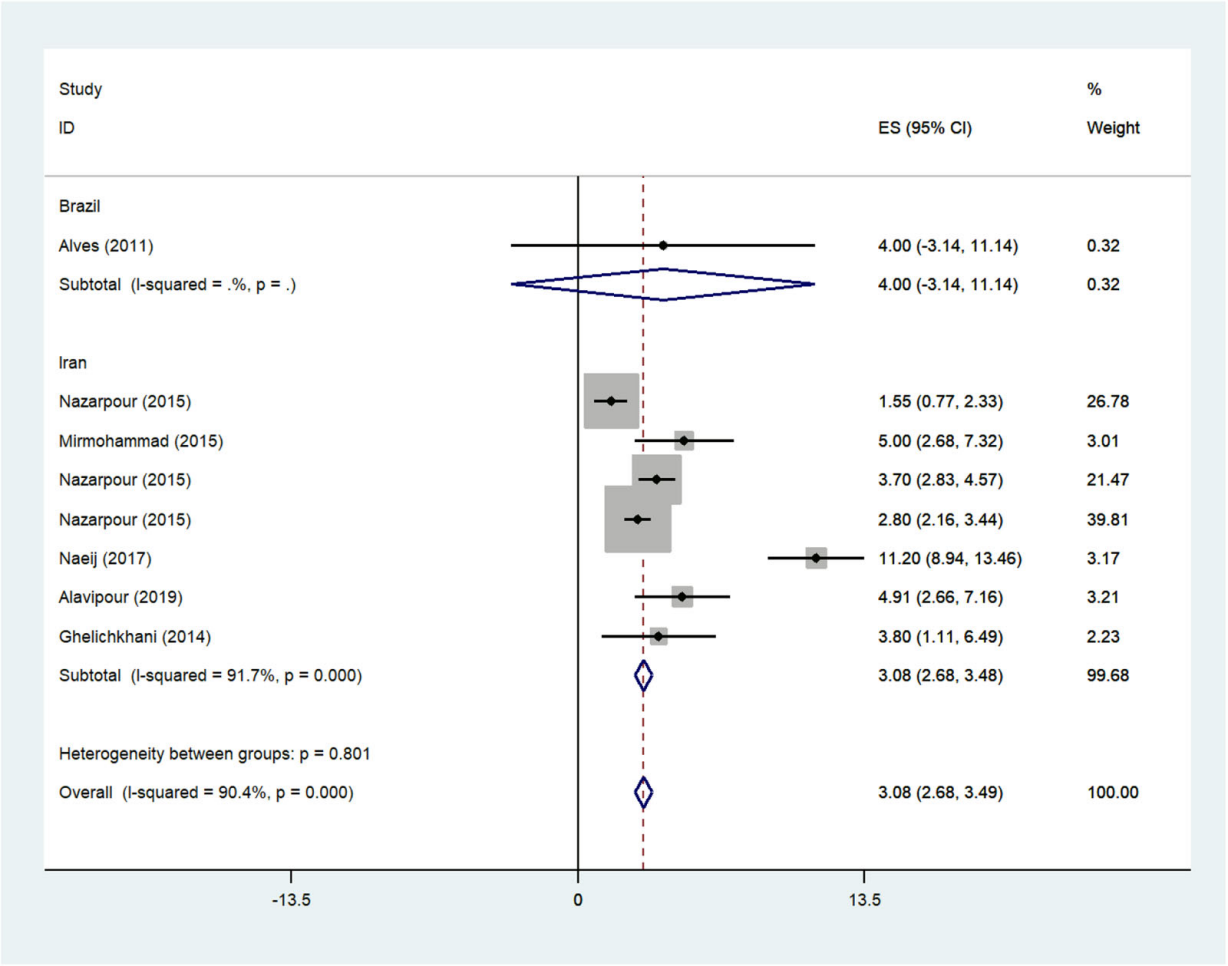
Forest plot for the intervention outcome. I^2^ is the ratio of the total variation due to heterogeneity, *p* > 0.05: heterogeneity is appropriate for interpretation, and ES shows effect size.

The meta‐analysis for eight intervention studies showed that the average difference between the two intervention and control groups was 3.08 (2.68–3.49), which was significantly different from zero (*p* < 0.001). The I² statistic was notably high at 90.4% (*p* < 0.001), indicating significant heterogeneity among the fixed‐effects results (Figure 6). To further investigate the source of this heterogeneity, a meta‐regression analysis was conducted. Meta‐regression analysis showed that study location (*p* = 0.41), time of study (*p* = 0.33), intervention duration (*p* = 0.45) was not the origin of heterogeneity and had no significant impact on the overall effect size. Egger's test and the relevant funnel diagram were drawn, and the results of this test showed that publication bias did not occur (*p* = 0.14). However, due to the asymmetry of the funnel plot and small sample size, the trim and fill method was also used to test the publication bias, and its result showed an insignificant publication bias (*p* = 0.064).

Likewise, individual studies indicated that the results from Alves and Naeij's results were significant outliers compared to the other studies. This result could confirm that Alves and Naeij's studies on postmenopausal women has a substantial impact on the overall ES of the intervention‐ studies. The result of the funnel plot showed a large deviation in sexual function outcomes between Alves and Naeij's studies and the other studies (Figure 4). It seems that this analysis lacked sufficient power to measure the impact of the intervention on the modification of sexual function due to the limited number of intervention studies, variations in program designs, low quality of research, and inconsistencies in the interventions programs.

## 4 Discussion

Postmenopausal women experience various changes, both physically and emotionally. Such changes can impact the quality of their social, family, interpersonal, and overall life relationships [59]. A significant complication in postmenopausal women is the decline in sexual function, which can lead to various changes in their quality of life [60, 61, 62]. Consequently, we aim to review the status of sexual function in postmenopausal women on a global scale to better understand how the menopausal transition affects sexual dysfunction and the sociodemographic factors that may be associated with this period.

### 4.1 Sexual Function Status

As shown in the systematic review, 75% of the total studies reported sexual dysfunction among postmenopausal women [1, 15, 17, 18, 19, 20, 21, 23, 24, 25, 28, 35, 36, 37, 38, 39, 40, 41, 42, 43, 44, 45, 46, 47, 48, 49, 50, 51, 52, 53, 54, 55, 56], primarily associated with a decline in the level of arousal, desire, and orgasm domains [1, 15, 17, 18, 19, 20, 24, 25, 28, 35, 36, 37, 39, 42, 43, 44, 46, 47, 50, 51, 52, 53, 56, 58]. This result underscored the importance of improving arousal, desire, and orgasm in these groups of women. In the meta‐analysis of 21 cross‐sectional studies with 9262 participants, the average sexual function score was below the FSFI cutoff of 26.55, indicating sexual dysfunction. Therefore, sexual dysfunction is a growing global concern and is prevalent in both developed and developing countries. This decline in various aspects of sexual function during menopause was associated with hormonal changes, specifically the reduction of estrogen hormone [60, 61, 62, 63]. However, many factors can affect sexual function during this phase of life, such as chronological aging, a woman's level of sexual functioning, length of the relationship, psychological and physical health status, partner's health and medication usage, educational level, and woman's feelings towards her partner [1, 15, 16, 17, 18, 19, 20, 21, 22, 23, 24, 25, 28, 35, 36, 37, 38, 39, 40, 41, 42, 43, 44, 45, 46, 47, 48, 49, 50, 51, 52, 53, 54, 55, 56, 57, 58].

The meta‐analysis results indicated no significant correlation between sexual function or dysfunction and geographical location or conditions. While some studies reported that environmental factors may influence sexual function, Chedraui et al. (2012) reported a contrasting finding, suggesting that residing at high‐altitude locations (3,500 to 5,500 meters above sea level) significantly impacts sexual function [63]. This effect may be attributed to the formidable challenges posed by human physiological changes that occur at elevated altitudes, such as low oxygen availability, high atmospheric pressure, extreme temperatures, intense ultraviolet radiation, and hypoxia. These changes can affect libido and sexual health outcomes. Therefore, the geographical context, particularly high‐altitude locations, plays a crucial role in understanding variations in sexual health outcomes [64, 65].

Although the meta‐regression analysis showed that study location, study period, participants’ education level, and age did not contribute to heterogeneity, the systematic review highlighted differences in sexual function levels associated with education level [17, 21, 40] and age [16, 22, 28, 39, 42, 46, 47, 48, 50, 52, 53, 57].

A systematic review revealed that 24% of the includec studies examined identified age as a significant determinant influencing various aspects of sexual health during menopause, including desire, arousal, orgasm, and the frequency of sexual intercourse within intimate relationships [21, 23, 24, 25, 40, 45, 48, 57]. Despite the importance of sexual function in postmenopausal women, sexual dysfunction increases with age. So, the decrease in sexual desire in postmenopausal women is much higher than in premenopausal women [63]. Dinerstein et al. (2001) observed significant declines in sexual function when comparing women who remained premenopausal to those transitioning from perimenopause to postmenopause [60, 61, 62]. This decline in sexual function may be due to hormonal fluctuations and the consequences caused by aging [12, 13].

The results showed that only 8% of the total studies in the systematic review measured the relationship between sexual function and education level and reported a significant positive relationship between sexual function and education in all dimensions. The level of education was one of the variables that affect the overall score of sexual function [17, 21, 40]. A study conducted by Orhan et al. (2019) on 310 menopausal women showed that a low level of education was one of the factors that increase the risk of sexual dysfunction. In this way, women with primary or low education were more likely to experience sexual dysfunction compared to those with a high school education or higher [66]. Marván et al. (2017) reported that women with higher levels of education are better at recognizing changes in sexual function and understanding its association with hormonal variations during menopause. These women are often more knowledgeable about management strategies, such as hormonal treatments, nonhormonal options, and therapeutic interventions. They also tend to possess better communication skills, which enable them to engage in open discussions with their healthcare providers and partners, ultimately fostering a more supportive environment [12, 13].

Limited studies indicated a significant relationship between the correlation between sexual dysfunction and family income [39, 47, 48, 52]. In 2019, Peixoto et al reported that women from middle or high socioeconomic classes had a higher likelihood of favorable sexual function. However, some Iranian studies have shown that individuals with high and middle incomes tend to have significantly lower sexual function because these women often experience increased job stress and responsibilities, which can negatively affect sexual desire and performance. Longer working hours leave less time for emotional intimacy, a crucial component of a healthy sexual life [47, 52].

The effect of sociodemographic factors on sexual function could not be conclusively determined due to limited studies with small sample sizes [17, 21, 40]. Therefore, caution should be exercised when interpreting these results. Considering the wide range and variability of sociodemographic characteristics influencing sexual function, it is essential to conduct longitudinal original studies to recognize their impact.

### 4.2 Intervention Effect

One of the primary research questions is to evaluate the evidence regarding the impact of interventions programs on the improvement of sexual function in postmenopausal women. The meta‐analysis showed that these interventions had a significant effect, with an average difference of 3.08 between the intervention and control groups. This indicates that the intervention programs notably enhanced sexual function in the postmenopausal women who participated in the intervention group [15, 18, 20, 56].

Based on the results of a systematic review, four studies showed a significant improvement in sexual function for the intervention group compared to the control group [15, 18, 20]. The intervention programs, which included training protocols, Question and Answer (Q&A) sessions, sex counseling workshops, and midwife‐based counseling, played a crucial role in enhancing women's skills and knowledge. These programs helped participants better understand management strategies for sexual health issues, the side effects of sexual dysfunction, available treatment options, and ways to improve overall sexual well‐being [18, 19, 43, 56].

Our findings showed that interventions based on a specific training protocol, a question‐and‐answer session, and a sex counseling workshop had a significant effect at the 0.001 level on improving outcomes [15, 20, 56]. Research indicated that women participating in tailored educational interventions or counseling workshops feel significantly more empowered to manage their sexual health. These programs enhance their confidence in discussing their needs with partners and healthcare providers by providing accurate information and fostering open dialog. These educational programs empower them in terms of sexual health by offering accurate information and openly addressing the issue [60, 61, 62]. These women understand hormonal fluctuations that affect sexual function and learn various treatment options, including medical treatments, lifestyle changes, and sexual health products that can improve their sexual experience. Through this intervention program, women can develop open communication and acquire comprehensive knowledge, which is necessary to adjust and manage their sexual well‐being in personal relationships [15, 20, 58].

Three studies revealed that intervention programs based on PEP, PFM, and Kegel exercises may not significantly improve sexual function in menopausal women, as not all women respond similarly to PFM and PEP. Factors such as medical conditions (e.g., hormonal imbalances and chronic pain), age, and psychological states (e.g., depression, anxiety, or low self‐esteem) can diminish the benefits of pelvic floor training and exercises [16, 18, 19].

The studies also emphasized that for physical activity programs to have a meaningful impact on women, these programs must be tailored to individual needs. When exercise programs lack sufficient duration and intensity, they may fail to deliver the desired improvements in sexual function. Furthermore, the research underscored the importance of emotional intimacy compared with intervention‐based physical activities. If these aspects are neglected, physical interventions may not influence the outcome [16, 19, 58]. Therefore, the impact of PEP, PFM, and Kegel exercises on sexual function in menopausal women can be limited by several psychological and medical factors. To enhance sexual health during this period, it is crucial to address these factors in a holistic manner.

### 4.3 Limitations

In this review, we aimed to provide a comprehensive, evidence‐based synthesis of research findings. However, certain common limitations may impact the applicability and validity of our conclusions. The variability in sample sizes, particularly the averaged percentage of sexual dysfunction may influence the generalizability of the findings. However, the meta‐regression analysis indicated that sample size did not significantly contribute to heterogeneity (*p* > 0.05) and had no notable impact on the overall effect size (ES) of sexual function or dysfunction across all studies. This underscores the importance of interpreting these results cautiously and within the appropriate context.

The limited literature and the small sample size may increase heterogeneity when assessing the relationship between sexual function levels and sociodemographic factors. Consequently, these relationships were only suggested in the systematic review and were not pooled in the meta‐analysis, which may have resulted in distorted overall findings. In intervention studies, limited research, variability in training programs, and low quality of interventions studies lead to significant heterogeneity when evaluating the effectiveness of intervention programs on improving sexual function. High variability complicated the synthesis and interpretation, making it challenging to draw generalizable findings. It is important to interpret these results with caution. However, Egger's test and trim and fill method showed that publication bias did not occur. Likewise, relevant studies in specific languages were excluded in this review. This may lead to language bias in understanding the topic. Another limitation is the delay in the submission of this manuscript, which may impact the timeliness of the findings. The study might not fully reflect the most recent trends that emerged after March 2022. Future studies will be important to ensure that the findings remain applicable and relevant in changing contexts beyond that date. Readers need to recognize these limitations when interpreting the combined conclusions of systematic reviews and meta‐analyses, as well as their relevance and validity for future research.

## 5 Conclusions

Despite its limitations, the quantitative synthesis of data on the status of sexual function‐associated factors in postmenopausal women has provided important insights for evidence‐based practice. The review reveals a significant prevalence of sexual dysfunction among postmenopausal women that are associated with adverse effects on sexuality, primarily linked to a decline in arousal, desire, and orgasm domains. Key demographic factors, such as age and education level, were found to substantially influence the risk of sexual dysfunction during menopause. This review emphasizes that implementing appropriate educational interventions plays a significant role in enhancing the knowledge of postmenopausal women to improve their sexual function within their relationships. From a practical standpoint, implementing well‐designed interventions with appropriate sample sizes and longitudinal and systematic follow‐up can enhance sexual function as a health factor in menopausal women. Likewise, sexual health as it relates to aging should be included in medical education and healthcare, with the focus on developing skills to cope with the social and psychological implications of sexual dysfunctions in postmenopausal women.

## Author Contributions

**Mohadese Hosseinabadi:** writing – review and editing, data curation. **Najmeh Khodadadi:** writing – review and editing, investigation. **Hadi Tehrani:** methodology, writing – review and editing. **Arezoo Orooji:** software, formal analysis, writing – review and editing. **Seyedeh Belin Tavakoly Sany:** writing – original draft, supervision.

## Conflicts of Interest

The authors declare that they have no competing interests.

## Availability of Data and Materials

The authors confirm that the data supporting the findings of this study are available within the article and its supporting information.

## Transparency Statement

The lead author Seyedeh Belin Tavakoly Sany affirms that this manuscript is an honest, accurate, and transparent account of the study being reported; that no important aspects of the study have been omitted; and that any discrepancies from the study as planned (and, if relevant, registered) have been explained.

## Supporting information


**Table S1:** details related to the inclusion and exclusion criteria. **Table S2:** Sexual function variation in interventional studies from baseline to follow‐up in control and intervention groups. **Table S3:** long version of Female Sexual Function Index (FSFI) questionnaire based on index domain scores. **Table S4:** Sexual function domains based on Female Sexual Function Index (FSFI) questionnaire.

## Data Availability

The data that supports the findings of this study are available in the supporting information of this article.
