# Supplementary material for: Sexual Function and Associated Factors in Postmenopausal Women: A Systematic Review and Meta‐Analysis
Source: Health Sci Rep. 2025 Oct 6;8(10):e71270. doi: 10.1002/hsr2.71270 (PMC12500535; doi:10.1002/hsr2.71270)
Supplement: Supplementary file 1 — Table S1: details related to the inclusion and exclusion criteria. Table S2: Sexual function variation in interventional studies from baseline to follow‐up in control and intervention groups. Table S3: long version of Female Sexual Function Index (FSFI) questionnaire based on index domain scores. Table S4: Sexual function domains based on Female Sexual Function Index (FSFI) questionnaire. [file HSR2-8-e71270-s001.doc]

**Table S1: details related to the inclusion and exclusion criteria**

| **Factors** | **Inclusion Criteria** | **Exclusion Criteria** |
| --- | --- | --- |
| **Participants** | Postmenopausal women |  |
| **Study Type** | Cross-sectional and interventional articles | Conference papers, fact sheet, grey literature (book, thesis.), and review articles |
| **Intervention** | All types of interventions were designed based on strategies to improve sexual function or dysfunction in postmenopausal women | Studies that lack of suitable effect size to show the effectiveness of intervention |
| **Setting and outcomes** | Report on sexual function scores and percentage of sexual dysfunction based on validate tools and its association with social demographic factors. |  |
| **Dissemination Type** | Full text articles published in indexed scientific journals |  |
| **Language** | English/Persian | Articles written in languages other than English/Persian |
| **Others** | No time and place limitations in the study |  |

**Table S2: : Sexual function variation in interventional studies from baseline to follow-up in control and intervention groups**

| **Code** | **Country** | **Sample size** | **Duration of intevention** | **h** | **Method** | **Tools and Material** | **Before Intervention**  **(Baseline)** | | **After intervention**  **(Follow-up)** | | Improvement level in intervention group | **Intervention Effect ^α^** |
| --- | --- | --- | --- | --- | --- | --- | --- | --- | --- | --- | --- | --- |
|  |  |  |  |  |  |  | intervention | Control | intervention | Control |  |  |
| 30* | Brazil | 32 | Twice per week for 12 weeks | 24 | PEP, training protocol | PowerPoint/ videos | 64.3±21.0 | 63.2±19.0 | 69.3±20.0 | 64.2±18.5 | 5 | P = 0.053 |
| 31 | Iran | 97 | Per week, 2-h | 11 | PFM exercises | telephone calls, educational booklets, Checklists for exercise | 22.46±0.60 | 23.16±0.40 | 23.52±0.55 | 22.67±0.330 | 1.06±0.02 | P= 0.167 |
| 32 | Iran | 100 | Four 2-h once a week | 25 | Training protocol | Educational booklet. | 17.47±5.5 | 17.77±3.5 | 22.47±4.5 | 17.77±3.5 | 5.02±0.02 | P<0.05 |
| 33 | Iran | 145 | Daily exercise, 2-h | 20 | Interview, Kegel exercises | Telephone calls, videos, booklets, images, checklists | 21.50±0.45 | 21.83±0.55 | 24.50±0.50 | 21.13±0.62 | 3.6±0.9 | P=0.05 |
| 34 | Iran | 104 | Per week, 2-h | 27 | Interview,  training protocol | PowerPoint, educational booklets, telephone calls | 21.41±0.52 | 20.91±0.32 | 24.33±0.52 | 21.11±0.22 | 3.02±2.87 | P<0.05 |
| 35 | Iran | 52 | _ | 20 | Midwife-based counseling, Q&A | ---- | 17.20±3.93 | 18.10±2.13 | 28.20±4.43 | 17.90±2.67 | 11.15±0.11 | P<0.001 |
| 36 | Iran | 90 | 4 - sessions of 45-60 minutes | 17 | Sex counseling workshop, Q&A | training booklet | 17.73±4.07 | 17.13±3.27 | 23.70±3.67 | 18.19±2.97 | 6.32±0.65 | P<0.001 |
| 37 | Iran | 100 | Once a week | 8 | Training protocol | Telephone calls, educational booklet | 19.29±5.25 | 20.09±4.25 | 22.09±4.25 | 19.09±5.19 | 2.47±2.07 | P=0.87 |

Personal exercise program (PEP); pelvic floor muscle (PFM); Question and Answer (Q&A), h= hour, All studies used long version of Female sexual function index (FSFI) questionnaire to measure sexual function except studies 30 that used SPEQ

**Table S3: long version of Female Sexual Function Index (FSFI) questionnaire bas**ed on index domain scores

| **Domain** | **Questions** | **Score Range** | **Factor** | **Minimum Score** | **Maximum Score** |
| --- | --- | --- | --- | --- | --- |
| **Desire** | 1,2 | 1-5 | 0.6 | 1.2 | 6 |
| **Arousal** | 3,4,5,6 | 0-5 | 0.3 | 0 | 6 |
| **Lubrication** | 7,8,9,10 | 0-5 | 0.3 | 0 | 6 |
| **Orgasm** | 11,12,13 | 0-5 | 0.4 | 0 | 6 |
| **Satisfaction** | 14,15,16 | 0 (or 1)-5 | 0.4 | 0.8 | 6 |
| **Pain** | 17,18,19 | 0-5 | 0.4 | 0 | 6 |
| **Full Scale Score Range** | | | | 2 | 36 |

A total score ranges from 2 to 36, with a commonly used cutoff score of 26.55 to identify women at risk for sexual dysfunction.

**Table S4:** **Sexual function domains based on Female Sexual Function Index (FSFI) questionnaire**

| **Code** | **Desire** | **Arousal** | **Lubrication** | **Orgasm** | **Satisfaction** | **Pain** | **Total score of sexual function** | **Comparison of domains** |
| --- | --- | --- | --- | --- | --- | --- | --- | --- |
| **1** | 2.06±0.84 | 2.51±0.90 | 2.47±0.96 | 2.65±0.72 | 3.11±0.70 | 0.85±0.81 | 13.65±4.93 | S>O>A>L>D>P |
| **2** | 2.82±1.40 | 3.10±1.55 | 3.31±1.78 | 3.11±1.73 | 3.72±1.50 | 3.25±1.73 | 19.31±9.69 | S>L>P>O>A>D |
| **3** |  |  |  |  |  |  |  |  |
| **4** | 2.1 ± 1.06 | 1.2 ± 1.4 | 1.5 ± 1.8 | 1.3 ± 1.8 | 1.9 ± 2.2 | 2.5 ± 2.9 | 10.5±11.16 | P>D>S>L>O>A |
| **5** | 3.80±0.93 | 2.85±1.10 | 4.39 ± 1.38 | 4.13±1.33 | 4.54 ± 1.22 | 4.39±2.64 | 24.11±6.04 | S>P>L>O>D>A |
| **6** |  |  |  |  |  |  |  |  |
| **7** |  |  |  |  |  |  |  |  |
| **8** |  |  |  |  |  |  |  |  |
| **9** | 4.2 | 5 | 5.2 | 4.1 | 5.1 | 5 | 28.7 | L>S>A=P>D>O |
| **10** |  |  |  |  |  |  |  |  |
| **11** | 2.77±1.55 | 2.72±1.91 | 2.90±1.93 | 3.08±2.10 | 3.57±1.76 | 3.18±2.14 | 18.22±10.60 | S>P>O>L>D>A |
| **12** | 2.4±1.0 | 1.3±1.7 | 1.4±1.9 | 1.6±2.0 | 1.8±2.2 | 1.8±2.2 | 10.1±10.3 | D>S=P>O>L>A |
| **13** |  |  |  |  |  |  |  |  |
| **14** | 2.92±1.09 | 2.95±1.22 | 4.04±1.48 | 3.73±1.33 | 4.23±1.08 | 4.73±1.43 | 22.6±6.64 | P>S>L>O>A>D |
| **15** | 3.02±1.34 | 3.58±1.69 | 3.96±1.88 | 3.63±1.93 | - | 4.29±2.34 | 18.48±9.18 | P>L>O>A>D |
| **16** | - | - | - | - | - | - | 18 ± 3.4  18.5 ± 3.3 |  |
| **17** |  |  |  |  |  |  |  |  |
| **18** | 43.3±27.2 | - | 52.8±33.4 | 50.6±38.8 | 50.3±26.0 | - | 53.3±29.3 |  |
| **19** |  |  |  |  |  |  | 22.35±8.87 | D>A>L=O>S>P  L>P>D>O>S>A |
| **20** | 2.32±1.02 | 1.69±1.54 | 1.89±1.91 | 1.81±1.79 | 2.99±1.97 | 2.60±2.39 | 13.31±9.15 | S>P>D>L>O>A |
| **21** | 2.6±1.1 | 2.7±1.5 | 3.3±1.8 | 3.1±1.8 | 3.4±1.6 | 3.6±2.0 | 18.8±8.7 | P>S>L>O>A>D |
| **22** | 2.68±0.86 | 2.75±0.89 | 2.77±0.85 | 2.81±0.93 | 3.73±1.14 | 4.13±0.96 | 18.92±4.25 | P>S>O>L>A>D |
| **23** | 3.80±0.93 | 2.85±1.10 | 4.39±1.38 | 4.13±1.33 | 4.54 ± 1.22 | 4.39±1.64 | 24.11 ± 6.04 | S>P>L>O>D>A |
| **24** | 2.64 ± 0.99 | 2.75 ± 1.06 | 3.42 ± 1.27 | 2.82 ± 1.03 | 2.70 ± 1.20 | 3.96 ± 1.10 | 17.93 ± 5.44 | P>L>O>A>S>D |
| **25** | 2.67±0.98 | 2.93±1.23  61.7% | 3.71±1.61 | 3.52±1.39 | 4.26±1.08 | 4.02±1.88 | 21.13 ± 6.02 | S>P>L>O>A>D |
| **26** | 2.07±0.9 | 2.25±1.89 | 2.07±1.35 | 2.01±1.55 | 2.07±2.05 | 3.09±1.19 | 28.09 ± 4.48 | P>A>S>L>D>O |
| **27** | 2.85±0.98 | 3.32±1.45 | 4.05±1.22 | 3.77±1.25 | 4.38±1.08 | 4.06±1.32 | 22.43 ± 7.3 |  |
| **28** | 3.12±0.97 | 3.26±1.38 | 3.09±1.17 | 3.12±1.21 | 3.32±1.15 | 3.37±1.38 | 19.28 ± 7.26 | P>S>A>O>D>L |
| **29** | 2.96±1.11 | 3.25±1.35 | 4.03±1.29 | 3.85±1.26 | 4.40±1.05 | 4.02±1.36 | 22.53 ± 5.91 | S>L>P>O>A>D |
| **30** |  |  |  |  |  |  |  |  |
| **31** | 3.57±0.11 | 3.10±0.12 | 3.77±0.16 | 4.36±0.15 | 4.84±0.13 | 3.93±0.16 | 23.52±0.55 | S>O>P>L>D>A |
| **32** |  |  |  |  |  |  |  |  |
| **33** | 3.92 ± 0.11 3.56 ± 0.11 | 3.38 ± 0.12 3.15 ± 0.13 | 4.15 ± 0.15 3.83 ± 0.15 | 4.12 ± 0.14 4.43 ± 0.14 | 4.57 ± 0.12 4.88 ± 0.12 | 4.45 ± 0.17 4.02 ± 0.17 | 24.50 ± 0.50 23.81 ± 0.51 | S>P>L>O>D>A  S>O>P>L>D>A |
| **34** | 3.87 ± 0.11 | 3.35 ± 0.13 | 4.13 ± 0.15 | 4.09 ± 0.14 | 4.50 ± 0.11 | 4.50 ± 0.19 | 24.41 ± 0.52 | P>S>O>L>D>A |
| **35** | 2.52 ± 0.81 | 2.61 ± 1.08 | 2.79 ± 0.92 | 2.91 ± 1.30 | 3.58 ± 1.16 | 2.64 ± 1.14 | 28.20 ± 4.43 | S>O>L>P>A>D |
| **36** | 3.18 ± 0.81 | 3.54 ± 0.76 | 3.80 ± 0.93 | 3.56 ± 0.97 | 4.62 ± 0.90 | 4.97 ± 0.56 | 23.70 ± 3.67 | P>S>L>O>A>D |
| **37** |  |  |  |  |  |  | 22.09±4.25 |  |
